# Prevalence of Sleep Disturbances in Pediatric Cancer Patients and Their Diagnosis and Management

**DOI:** 10.3390/children8121100

**Published:** 2021-11-29

**Authors:** Irtiza N. Sheikh, Michael Roth, Peter L. Stavinoha

**Affiliations:** Division of Pediatrics and Patient Care, The University of Texas MD Anderson Cancer Center, 1515 Holcombe Blvd., Houston, TX 77030, USA; mroth1@mdanderson.org

**Keywords:** sleep disturbance, insomnia, excessive daytime sleepiness, pediatric cancer, survivorship, adolescents and young adults

## Abstract

Sleep disturbances represent an understudied yet common source of distress among pediatric cancer patients and survivors, with deleterious effects on quality of life. Sleep issues stem from multiple risk factors, yet individual contributors are difficult to isolate, consequently impeding the identification of targets for intervention. In many pediatric cancer patients, disrupted sleep and its negative impact on quality of life continue into adulthood and may affect various functional domains. This literature review highlights the types and prevalence of sleep disturbances in pediatric cancer patients during active treatment and through survivorship. Potential etiological and risk factors for disturbed sleep are summarized, including the effects of cancer and its treatment, psychosocial and family factors, as well as individual-patient aspects, such as genetics, mood and coping skills. While existing assessment and management strategies are reviewed, the literature is incomplete, and significant gaps emerge in our understanding of sleep disturbances in pediatric cancer patients and survivors. The review concludes with recommendations of areas where further research is needed. The aims of this review include increasing clinicians’ awareness of sleep disturbances as a significant source of poor quality of life in pediatric cancer patients and survivors and directing researchers to gaps in our understanding of sleep disturbances in pediatric cancer patients and survivors.

## 1. Introduction

**Figure 1:**
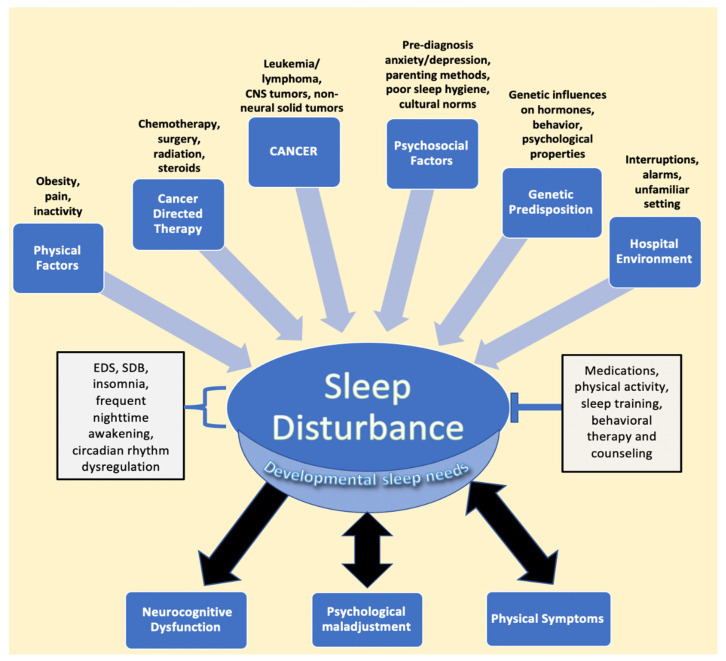
Factors impacting sleep in children diagnosed with cancer. The figure describes the various entities described in the literature that lead to sleep disturbance, followed by the resulting sequalae of negatively impacted sleep. EDS, SDB, frequent nighttime awakening, and circadian rhythm dysregulation are components of disturbed sleep, while interventions such as medications, physical activity, sleep training, and counseling represent mitigating factors that relieve sleep issues. Developmental sleep needs are affected globally by sleep disturbances, despite the fact that children of different ages require different amounts of sleep. Psychological maladjustment and physical symptoms, such as pain, have been found to have a bidirectional relationship with sleep issues. Abbreviations: CNS, central nervous system; EDS, excessive daytime sleepiness; SDB, sleep disordered breathing.

Cancer in the pediatric and adolescent population (1–19 years old) remains one of the three leading causes of death in the United States [1]. However, advances in the treatment of pediatric cancer have led to significant increases in long-term survival, highlighting the need to address not only survival but treatment-associated morbidity and improved quality of life as well [2,3]. Among quality-of-life manifestations, children, adolescents, and young adults report sleep disturbances during and after treatment [4,5,6,7]. Indeed, sleep issues and their sequelae appear to be common in pediatric cancer patients and survivors, with almost half experiencing some form of sleep disturbance [8,9]. The most common etiologies of poor sleep in patients and survivors include increased daytime sleepiness and insomnia, followed by increased time to sleep onset, decreased sleep duration, and overall poor sleep quality, issues that may be manageable or treatable [8,9,10,11]. Sleep disturbances may directly result from cancer and its treatment, as well as from multiple other risk factors, many of which lead to a bidirectional relationship between sleep and physical and psychological distress [12,13,14]. This multifactorial relationship between pediatric cancer and sleep disturbances is illustrated in Figure 1. 

The harmful effects of non-restorative sleep on the quality of life of pediatric cancer patients remain an understudied aspect of cancer care and survivorship [12]. The goal of this integrated review was to synthesize the literature on sleep disturbances in patients and survivors of childhood cancer, summarize known and suspected etiologies, highlight potentially fruitful intervention paradigms, and identify areas where further research is needed. The review begins with an overview of the direct effects of cancer and treatments on sleep, followed by environmental and psychological factors that may affect sleep quality. Finally, common sleep assessments and interventions are reviewed, followed by recommendations for further research to refine the clinical identification and management of sleep disturbances in this population.

## 2. Methodology

This literature review represents a scoping review in which the question identified was in regard to the current breadth of literature as it relates to the prevalence, cause, diagnosis, and treatment of sleep disturbances in cancer. We then identified the peer-reviewed literature that was relevant to the question, using PubMed, MEDLINE (Ovid), and Google Scholar databases. Examples of search terms that were used included, “sleep issues in pediatrics”, “sleep disturbances in pediatric cancer”, “sleep disturbances in pediatric cancer survivorship”, “diagnosing sleep issues in pediatric patients with cancer”, “treating sleep disorders in pediatrics”, and “treatment of sleep issues in pediatrics”. In order to describe sleep issues during treatment, as well as survivorship, this review considered studies that included children and adolescents who had been diagnosed between the ages of 0 to 18 years old. Studies that did not include patients with a history of pediatric cancer were excluded. In terms of data that described the management of sleep issues in pediatric cancer patients, we included studies that evaluated pharmacological and non-pharmacological interventions in pediatric and adolescent patients, regardless of their cancer status. Studies describing survivorship and sleep issues in survivorship included patients of all ages, including adults. Studies that described the tools and surveys used to diagnose patients with sleep disorders were included if they involved pediatric and adolescent patients, regardless of cancer status.

## 3. Clinical Implications of Sleep Issues

The importance of sleep in developing children cannot be overstated. Sleep represents a complex, multi-staged process required for survival, daily functioning, neurocognitive and physical development [15]. Moreover, per the American Academy of Sleep Medicine, developmental sleep needs change as children get older, generally requiring lesser amounts of sleep in adolescence.

Poor sleep in children is associated with deleterious effects on mood and neurocognition; a higher risk of clinical depression and anxiety; increased behavioral difficulties, including increased rates of hyperactivity; and less success in school [13,14,16,17]. In terms of physical health, children with sleep deficiencies or delayed sleep onset times have been found to be at higher risk of obesity and chronic health problems, such as hypertension and diabetes [18,19,20]. There also exists a reciprocal relationship between sleep and pain in which pain leads to disturbed sleep and poor sleep heightens the perception of pain, a relationship that is understudied in pediatric cancer patients and survivors [12,21,22]. 

Cancers such as leukemia are most common in those between the ages of 0–14 while central nervous system (CNS) tumors are more commonly seen in ages 15–19 [23]. Considering that certain cancers are more prevalent in different age groups than others and may affect sleep differently, the types of sleep disturbances more commonly observed in each age group may differ as well. However, excessive daytime sleepiness (EDS), a form of hypersomnolence that makes it difficult for children and adolescents to stay awake during daytime activities, is one of the most common ramifications of sleep disturbances in pediatric cancer patients, overall [15,24]. Compared to other types of sleep issues, EDS can have a similar impact on children’s mood, attention and performance in school and their cognition [8,25,26,27]. In fact, among younger cancer survivors compared to adolescents, sleep deprivation has a stronger effect on cognition, which indicates that disrupted sleep has a more deleterious effect on the younger, developing brain [28,29]. Regardless of cancer type, sleep disruption is associated with difficulties in multiple domains, including mood regulation, attention, academic performance, task efficiency, processing speed, and fatigue, as well as increases in risk-taking behavior, such as tobacco use [14,15,26,27,30,31,32,33,34,35,36,37,38]. These impacts on quality of life pose a challenge for clinicians and are a call to action for researchers to improve outcomes for pediatric cancer patients.

## 4. Relationship between Cancer and Treatment and Sleep in Pediatric Cancer

EDS and SDB are the most common sleep issues seen in patients and survivors of hematological malignancies, as well as solid tumors, such as sarcomas, impacting as many as 60% and 40% of patients, respectively, and they are even more common in those with CNS tumors [2]. While the direct role that these cancers play in causing sleep issues is unclear, sleep disturbances occur past the treatment phase, at times more than 5 years after treatment, as demonstrated in a study by Clanton et al. of adult survivors of childhood cancer [14]. Chemotherapy, steroids, and radiation therapy are the mainstays of pediatric cancer therapy and also play a significant role in altering sleep patterns [39]. Changes in sleep during treatment have been observed in a wide array of malignancies, including non-neural solid tumors, hematological cancers, and CNS tumors, despite the differences in the specific chemotherapies administered [39,40]. Hospitalization is also a component of treatment in which initial or subsequent admissions have been shown to negatively affect sleep in pediatric cancer patients [41,42]. Although patients treated in the outpatient setting tend to have better sleep quality than those in the inpatient setting, they still have an elevated risk for poor sleep quality during active treatment. This is evidenced in a study by Zupanec et al., where greater than 80% of ALL patients between the ages of 4 to 18 years old on outpatient maintenance chemotherapy described poor sleep characterized by increased nighttime awakenings and wake time after sleep onset when compared to pediatric norms [40]. 

In the sections below, the effects of cancer on sleep during treatment and into survivorship are explored, followed by a discussion on the role treatment itself plays in contributing to sleep issues in patients and survivors. 

### 4.1. Effects of Hematological Malignancies and Non-Central Nervous System (CNS) Solid Tumor Cancers on Sleep

Nearly 40%, and, in some cases, greater than 80%, of pediatric leukemia patients report some form of sleep disturbance, with insomnia being the most prevalent; however, there is a need to describe the direct impact of pediatric health conditions, such as cancer, on sleep [2,40,43,44]. Researchers have explored the biology of the circadian clock in leukemia cells and propose that alterations in the circadian rhythm of cells allow for unchecked replication [45,46,47,48,49]. There is a need for further research to determine the direct effects of hematological malignancies on sleep; the relationship between circadian gene expression in cancer cells and circadian-rhythm-dysregulation-related sleep disruption may represent a starting point for such an investigation. 

In contrast to hematological malignancies, non-neural solid tumors may play a more direct and measurable role in causing sleep disturbances. They can result in OSA or SDB because of their anatomic location relative to the upper and lower airways and may necessitate surgical removal to relieve the obstruction [50]. However, outside of a case report of a spinal osteochondroma leading to sleep apnea or studies with mixed cancer diagnoses, no significant studies have isolated the role of pediatric sarcomas in sleep issues [10,50,51]. Nunes et al. presented one of the few studies that recognized that patients with sarcoma have greater difficulty with sleep and fatigue, possibly due to associated bone pain [52]. 

In pediatric leukemia survivorship, there is wide variation in the reported prevalence of sleep disturbances, with rates ranging from 13% to 50% in acute lymphoblastic leukemia survivors [51,53]. These differences in rates are compounded by reports of pediatric leukemia survivors who do not experience clinically poorer sleep than their sibling controls [51,54]; this contrasts with reports that indicate that leukemia survivors report disturbed sleep more than a decade after treatment [53]. Conflicting data on sleep in leukemia survivors lack a satisfactory explanation and highlight the need for further research to discover the true changes in sleep. 

Similar to its hematological counterpart, the effects of lymphoma on sleep in survivorship represent another opportunity for further investigation. Research from the Childhood Cancer Survivor study looking at the factors that influence fatigue and poor sleep in adult survivors of childhood Hodgkin lymphoma (HL) demonstrated that survivors with a high body mass index (BMI) and significant bodily pain were more likely to experience EDS [55]. In that study, those with at least “medium” pain were five times more likely to experience poor sleep quality. In reviewing the effect of BMI on sleep, the effect on HL survivors is similar to those of obese survivors of CNS tumors, with higher BMI scores correlating with higher risks of sleep issues [56]. This highlights that BMI is a predictive factor that affects the sleep quality of lymphoma survivors and may be extrapolated to be a cause of disturbed sleep in other survivors, regardless of cancer type.

There is a significant lack of research on the direct effects of non-CNS cancers on sleep in both patients on treatment and those in survivorship. The results of current studies highlight that non-CNS cancers can directly impact patients’ physiological, psychosomatic, and anatomical features to disrupt sleep. Each point of disruption, such as pain, BMI, and physical-activity limitations, may serve as a modifiable target for interventions to improve sleep quality in patients and survivors [55,57,58,59,60,61]. Moreover, as further explained below, the type of cancer and its location may help clinicians predict the various types of sleep disturbances in these individuals.

### 4.2. Effects of CNS Tumors on Sleep

Nearly 80% of patients with CNS tumors experience sleep issues, with EDS being the most common in survivorship, as described in a study by Rosen et al., where greater than 60% of CNS tumor patients experienced EDS in survivorship [2,32]. Those with tumors of the brainstem, thalamus, or hypothalamus are the most frequent cancer patients referred for sleep studies; brain tumors are the second leading cause of secondary narcolepsy [62]. More than a quarter of survivors of pediatric brain tumors are also noted to suffer from insomnia [63]. However, research into the causes of sleep disturbances in pediatric CNS tumor patients is lacking compared to that in pediatric CNS tumor survivors. Examples indicate that the location of CNS tumors and disruption of neural structures that regulate sleep through hormonal control are the strongest examples of the direct effects of brain tumors on sleep [64,65,66]. Craniopharyngioma, a relatively common pediatric brain tumor that typically affects the hypothalamus, can disrupt melatonin secretion due to damage to the suprachiasmatic nucleus, leading to daytime sleepiness and poorer quality of life [67,68,69,70]. Posterior fossa tumors, including those at the brainstem, can result in apneic episodes during sleep, narcolepsy, or SDB, due to infiltration of respiratory centers, leading to EDS [2,62,71,72,73]. Interestingly, tumors in areas that are not ordinarily associated with sleep, such as the cerebellum, can lead to sleep disturbances, with potentially fatal consequences stemming from respiratory failure due to central sleep apnea, indicating that the cerebellum, primarily thought to direct motor function, may also have a role to play in controlling respiration, and the disruption of that respiration control leads to central sleep apnea [65,71,74]. Moreover, as Lee et al. describe, the effects of therapies, such as chemotherapy and radiation, on the cerebellum in contributing to sleep apnea is unclear but is probably a contributing factor to the observed central sleep apnea [74]. The effects of various pediatric CNS tumors indicate that, taking into account different locations and comparing their impact on sleep during treatment represent fruitful areas of investigation. This is especially important in pediatric oncology, considering that medulloblastomas, which occur in the cerebellum, are among the most common CNS tumors in children [75]. 

In adult survivors of pediatric CNS tumors, EDS continues to remain the main source of sleep disruption, followed by issues such as increased sleep onset latency, insomnia, and SDB [2,6,26,32,33,39,62,63,67,68,76,77,78]. Moreover, CNS tumor survivors with high BMI scores had lower levels of melatonin and were at a higher risk of hypersomnia, narcolepsy, and SDB, indicating that hormonal disruption continues into survivorship and is a significant component of sleep disturbance, especially in those with high BMIs in the range of obesity [51,68,76,79]. 

While associations have been documented between EDS and brain tumors in the pediatric cancer population, the etiologies of certain sleep disturbances are not always clear. For example, resolution of SDB does not always lead to an improvement in EDS, suggesting that EDS is a multifactorial problem due to inefficient sleep, significant night awakenings, or circadian rhythm disorders that may manifest from varying sources [78,80]. 

Overall, the literature examining the impact of brain tumors on sleep indicates that the location of a brain tumor, its effect on hormone disruption, and its structural disruption of the brain contribute to sleep disturbances [67,69,71,74]. Moreover, EDS is a significant source of distress in patients and can result due to radiation, chemotherapy, or surgery, and as Rosen et al. demonstrate, it can continue into survivorship [32]. Pediatric CNS tumor patients and survivors represent an important population for research into the direct effects of cancer on sleep, especially as pediatric CNS tumors are the most frequent pediatric cancer referred for sleep studies [2]. 

### 4.3. Effects of Chemotherapy on Sleep

Patients undergoing chemotherapy report significant sleep disturbances, characterized by increased nighttime awakenings and restlessness [40,81,82]. In a sample of mainly leukemia and non-Hodgkin lymphoma patients, 95% of adolescents undergoing chemotherapy experienced disrupted or low-quality sleep at least three times a week that was associated with tiredness, decreased alertness, and decreased satisfaction with the prior night’s sleep quality [30]. 

In terms of specific risk factors and mechanisms, some patients who report sleep disturbances during chemotherapy have been found to have gene polymorphisms in genes encoding Interleukin-6 (IL-6) and tumor necrosis factor (TNF) that lead to elevated cytokine levels in the context of inadequate sleep [83]. As shown by Cheung et al. in a study looking at ALL survivors at a median of 7 years following diagnosis, the effects of chemotherapy on disturbed sleep, such as increased nighttime awakenings, may persist well into survivorship, as a result of an increase in cortisol, cytokines, and the associated physiologic cascade of immune and inflammatory responses [38,84]. These sleep issues have been shown to lead to worsened neurobehavioral outcomes, such as aggression, attention difficulties, and learning issues [85,86]. 

Chemotherapy also plays a role in increasing the risk of developing psychological disturbances associated with impaired sleep, including anxiety, mood disorders, and behavioral issues [38,84]. Further research is needed to identify the extent to which chemotherapy independently affects sleep during treatment and survivorship, as well as to improve our understanding of the underlying risk factors and mechanisms of sleep disturbances that result from chemotherapy [81]. 

### 4.4. Effects of Steroid Treatment on Sleep

Steroids such as dexamethasone and prednisone are commonly used in the treatment of cancer patients and have been found to contribute to insomnia in adolescent acute lymphoblastic leukemia patients, some of whom require sleep aids [2]. Dexamethasone, in particular, is associated with poorer sleep quality compared to those on prednisone, longer time spent napping to compensate for inefficient nighttime sleep than prednisone, and disruption of REM sleep [87,88,89,90]. As evidenced by a study in ALL patients between the ages of 5 and 18 years old, dexamethasone is also associated with changes in circadian rhythm activity and an increased feeling of fatigue [91]. Sanford et al. also demonstrated, through the evaluation of patients with ALL on maintenance treatment, that gender differences may exist in experienced sleep issues, considering that inadequate sleep duration and quality due to dexamethasone appeared to be exaggerated in females [92]. 

More research is needed to fully understand the mechanisms of sleep disturbance that result from steroid treatment and why some agents (e.g., dexamethasone) appear to have more impact than others (e.g., prednisone). The differing effects of the two steroids may be attributable to the nearly three-fold longer half-life, stronger potency, and increased CNS penetration of dexamethasone when compared to prednisone [93,94]. Further, in a study exploring the mechanism behind dexamethasone’s effects on sleep, pediatric leukemia patients on dexamethasone with certain genotypes for the genes *IL-6*, polymerase delta-interacting protein 3 (*POLDIP3*), or α_2_-Heremans-Schmid glycoprotein (*AHSG*), which encodes a hepatic protein, were affected most negatively in terms of sleep duration and efficiency, again indicating that patient-specific characteristics increase vulnerability to sleep difficulties under certain conditions and treatments [95]. 

There is a consensus that steroids lead to increased daytime napping and nighttime insomnia; as they are a mainstay of cancer treatment, practitioners should be aware of the sleep disruption that they can cause. As with chemotherapy, more research is necessary to ascertain the impact of individual steroid agents on sleep independent of other factors, as well as to better identify patient characteristics that may lead to greater vulnerability to sleep disturbance with steroids.

### 4.5. Effects of Radiation Therapy on Sleep

Few works in the literature exist regarding the direct effects of radiation therapy on sleep in pediatric cancer patients. Cranial radiation affects brain structures, such as the suprachiasmatic nucleus that regulates sleep and wakefulness, through direct injury to those structures [31,56,96,97]. Effects of such radiation-induced injuries to the sleep-regulating structures in the hypothalamic–pituitary axis, including the SCN, may persist into adulthood [56,76,96,97,98]. 

Endocrine changes secondary to radiation therapy may also have a role in affecting sleep years after treatment. For example, adults who were treated as children with cranial radiation therapy (CRT) and reported difficulty with wakefulness after sleep demonstrated lower growth-hormone peak levels after an insulin challenge [96]. Considering that the hypothalamus regulates growth hormone release and sleep, both functions that are potentially affected by CRT, further research is needed to determine the interplay not only between the disruption of growth hormone release due to CRT and sleep but the long-term impact of CRT on the brain structures involved in the hormonal control of sleep [99,100]. 

### 4.6. Effects of the Hospital Environment on Sleep

The hospital environment leads to shorter total sleep duration and difficulty remaining asleep during inpatient stays [41,81,101,102,103]. Hospitalized pediatric cancer patients with disrupted sleep experience greater levels of fatigue and tend to sleep longer during the day to compensate for nighttime disruptions that are at least partly related to nighttime room entries and exits, excessive lights, and noise [41,42,104]. When comparing specific cancers, Graef et al. described that hospitalized pediatric patients between the ages of 4 and 19 years old with medulloblastoma exhibited drastically shorter sleep durations and higher sleep onset latency than pediatric patients with ALL or non-CNS solid tumor patients [105]. Such studies highlight the potential for optimizing the inpatient environment and clinical practices as intervention targets to improve sleep in pediatric cancer inpatients. 

## 5. Psychological and Behavioral Factors That Affect Sleep

Inherent psychological states, such as temperament, ability to cope with the cancer diagnosis, mood, and behavior, can affect sleep following a cancer diagnosis; they are also factors that are affected by sleep disturbances themselves. Considering that nearly a quarter of pediatric cancer patients and survivors report clinical depression or anxiety, it is important to explore the bidirectional relationship between mood and sleep [106]. During pediatric leukemia treatment, a child’s temperament, coping skills, and self-calming skills are impacted by a cancer diagnosis and lead to an increase in psychological distress [44]. In these patients, psychological distress most commonly leads to difficulty falling asleep, awakening in the middle of the night, and irregular sleep; however, how the disrupted sleep alters the mood of such patients is unclear [44]. 

Although research into the bidirectional relationship between mood and disturbed sleep in pediatric cancer patients during treatment is lacking, research in those in survivorship is informative. In survivors, sleep disturbances contribute to the development of depression, post-traumatic symptoms, and anxiety, similar to other pediatric populations [27,31,53]. Insomnia is the most common sleep issue that has a strong bidirectional relationship with anxiety, a phenomenon that is also observed in survivors of childhood leukemia, as detailed by Zhou et al., wherein adult survivors of childhood cancer with insomnia experienced elevated anxiety levels [13,63,107]. Inversely, similar to mood disorders that impact sleep in non-cancer pediatric populations, large studies, such as those from the Childhood Cancer Study, have shown that depression in pediatric cancer survivors leads to an increased prevalence of sleep issues, such as increased EDS [31,108]. When analyzing the interplay of sleep disturbances and mood, a vicious cycle results in which sleep disturbances can represent both a contributor to, and a consequence of, psychological stress [27]. 

Hospitalization for cancer treatment is associated with factors such as anxiety during procedures and child and parental co-sleeping, which may continue in outpatient care and persist well after the completion of treatment [44,109]. Indeed, parental behaviors change as a result of a childhood cancer diagnosis [110,111,112]. Studies such as the one by McCarthy et al., regarding parents of pediatric ALL patients, have shown that co-sleeping is adopted by parents to alleviate both parental and child anxiety but is strongly associated with ineffective sleep [44,112]. Researchers such as McCarthy et al. and Simard et al. have described that parents’ learned behaviors, such as giving patients food and drink prior to bedtime and using comforting techniques, are factors that may exacerbate inefficient sleep [112,113]. 

Practitioners should be aware that mood, behavior, and the inherent psychological predisposition of the patient and caregiver play significant roles in sleep. However, there is a reciprocal relationship in which sleep also alters patients’ emotions and moods. Therefore, addressing these interconnected and multifactorial etiologies is important to breaking the cycle of disturbed sleep and mood.

## 6. Clinical Assessment and Measurement of Sleep Disturbances in Children with Cancer

When evaluating patients for sleep disturbances, clinicians must assess various components of sleep quality, a broad term that encompasses the amount of sleep, sleep latency, the need for sleep aids, and the extent to which sleep affects daytime functioning [114]. An assessment of sleep disturbances in children, with or without cancer, begins with a comprehensive history and physical examination [115]. Objective measures, such as BMI, evaluation of the oral cavity for tonsillar hypertrophy, and blood-pressure measurements, may help discover sequelae related to SDB and snoring [115]. Factors such as the use of television prior to sleep; family and academic pressure; and differing cultures, such as those where co-sleeping is encouraged or bedtimes are less rigid, must also be accounted for when determining contributors to inefficient sleep [115,116]. Measures such as polysomnography, actigraphy, sleep diaries, and sleep questionnaires serve as supplemental tools to the history and physical examination and provide a more complete picture of the source and type of sleep disturbances experienced. 

Polysomnography and wrist actigraphy represent two objective measures of sleep, while sleep diaries and surveys are influenced by subjective input. While polysomnography is considered the gold standard for diagnosing SDB and sleep limb movement disorders, it may not be feasible in some settings because of the expense, the need for a child-friendly sleep laboratory, and the inability to diagnose sleep disorders associated with behavior [115,117,118]. Wrist actigraphy provides sleep data through a continuous monitor that measures activity such as sleep/wake cycles and sleep efficiency, but it is poorly equipped to evaluate the stages of sleep [119]. When used alongside a sleep diary, the data from both modalities are effective at assessing sleep quality [118,120]. Although it is a subjective measure, sleep diaries maintained by the parent or child provide insight into sleep quantity and timing on a consistent nightly basis [115,120].

Self-reported questionnaires are imperfect but valuable and convenient tools to categorize and quantify the presence and severity of sleep disturbances [121]. As a testament to the usefulness of sleep surveys, the American Academy of Sleep Medicine used data from parent- and child-reported sleep questionnaires to develop consensus recommendations for sleep duration in children and adolescents [7]. A variety of sleep questionnaires, such as the Children’s Sleep Habits Questionnaire and Sleep Disturbance Scale for Children, are reliable surveys for assessing multiple components of sleep, including duration of sleep and disorders of initiating and maintaining sleep; they have also been used to measure sleep in studies of pediatric cancer patients [43,44,121,122,123]. However, the Patient-Reported Outcomes Measurement Information System pediatric sleep scale is the only measure that has been validated specifically in the pediatric cancer population [124]. 

The benefits of these surveys include their affordability and ability to evaluate a wide range of sleep disturbances through self-reported methods. However, in surveys reported by parents on behalf of patients, there was a risk of over- or under-reporting the severity of sleep issues [122,123]. Some questionnaires are also limited in the ages in which they have been studied and can be reliably used.

There are various objective and subjective tools available to clinicians to investigate sleep disturbances in the children. These tools can generally be used together to provide a more complete picture of sleep issues and their impact on daily functioning. However, there is a need to develop questionnaires and validity studies that specifically target pediatric cancer patients, while taking into account cancer-specific factors, such as treatment and cancer type. Regardless of their crude nature, sleep surveys are an efficient and valid way for clinicians to identify sleep disturbances that may lead to the faster initiation of intervention strategies to mitigate the negative functional and quality-of-life impacts of sleep problems in this population. Table 1 summarizes a variety of sleep-assessment methods for clinicians to use to evaluate pediatric cancer patients and survivors. 

## 7. Managing and Preventing Sleep Disturbances

There are no official guidelines on preventative or treatment methods for sleep disturbances in pediatric cancer patients and survivors; in addition, no approaches have been evaluated in large randomized controlled trials, and no systematic research has examined specific interventions associated with individual or combined risk factors [134]. Given the mosaic of potential etiologies for sleep disturbance in these patients, additional research is necessary to disentangle the independent contributions of factors, including cancer-specific risks, treatment-related factors, environmental and behavioral contributors, and psychological components. Regardless of modality, the importance of early intervention is underscored by the fact that sleep disturbances can continue into adulthood for childhood cancer survivors [11,106,135]. 

The Predisposing, Precipitating, and Perpetuating Model of Insomnia is a potentially useful model for identifying intervention targets for the sleep disturbances observed in pediatric cancer patients and survivors [27]. This model considers factors that predispose patients to insomnia or sleep disturbances, such as genetics and family history. The sleep disturbances are then precipitated by factors such as the stress of treatment, hospitalization, and the psychosocial and physiological changes that are associated with diagnosis and treatment [27,41]. Twin studies and isolation of mutations in genes, such as the transporter region of the serotonin gene (*5HTTLPR*), have demonstrated that genetic factors also predispose patients to an increased risk of emotional disturbance due to sleep issues, perpetuating the bidirectional relationship between sleep and emotional issues [13,136,137]. When patients return home from an inpatient stay, psychological and physical stress, such as constant anxiety, perpetuate maladaptive sleep habits that developed throughout inpatient treatment, including making it harder to fall asleep at home or experiencing nighttime awakenings [109]. The model represents points in the cycle that serve as preventative and intervention targets and indicates that effective treatment requires managing a multitude of factors, including the cancer itself, the side effects of treatment, and the psychosocial states of the patient and his/her family. 

Although no large randomized controlled trials have evaluated the management of sleep issues in the pediatric cancer population, small pilot studies have evaluated various modalities in the inpatient setting as potential interventions to manage and improve sleep, such as massage and physical activity [138,139]. Complementary and alternative methods (CAMs), such as massage, yoga, hypnosis, and acupuncture, have been used in adult and pediatric cancer patients, yet their effectiveness and safety in pediatric cancer patients have not been widely evaluated [134,140,141]. Such methods, especially non-pharmacological approaches, represent another avenue that may have a role in managing and preventing sleep issues in pediatric cancer patients, but there is a lack of current research on this issue [134]. 

Educating families on sleep hygiene (e.g., appropriate wake and sleep times and activities prior to sleep) has also been used to mitigate sleep issues during cancer treatment. On the basis of the understanding that sleep habits change during cancer treatment and parenting behaviors play a role in influencing patients’ sleep, investigators educated families of pediatric ALL outpatients on sleep issues and provided them with relaxation techniques to influence family behavior and promote good sleep hygiene. This program resulted in longer nighttime sleep duration and shorter wake time after nighttime awakening, indicating that education and relaxation techniques are viable management strategies in other pediatric cancer and survivor populations [106,142]. Moreover, while there are no established methods for preventing sleep issues in pediatric cancer patients, educating parents in the general population on normal sleep patterns in children is an effective method of avoiding more entrenched issues, such as insomnia, or identifying them earlier; such educational programs should be trialed more broadly in families of pediatric cancer patients [143]. 

Sleep and exercise may be reciprocally beneficial treatment paradigms for sleep disturbance, where an improvement in one can result in an improvement in the other [144,145,146]. This is supported by a study of adolescents with various types of cancers including hematological malignancies and solid tumors who participated in a program where physical activity during treatment was compared to those with lower levels of activity. The researchers demonstrated that those with greater frequency and duration of physical activity during treatment and survivorship experienced significantly improved sleep quality and in turn, directly improved quality of life [147]. Considering that BMI has been shown to serve as a risk factor for EDS in children, modifying dietary choices along with activity is also vital to mitigating the effects of sleep disturbances [51,78]. In a study of cancer patients between the ages of 8 to 18 years old who were actively undergoing chemotherapy, higher levels of daytime physical activity were shown to decrease waking after sleep onset and increase sleep efficacy [144]. Overall, increasing physical activity and decreasing BMI in the pediatric cancer population serve as direct methods to improve sleep quality and reduce the effects of poor sleep on functional outcomes. 

Various psychological therapies may hold promise for managing sleep disturbances in pediatric cancer patients. Adventure-based training is a combination of self-reflection techniques and sustained physical activity; in a small randomized controlled trial, it was shown to counter cancer-related fatigue [148]. Similar studies can be implemented to discover the role of adventure-based training in improving sleep in cancer survivors. Another psychological therapy that may have utility in the pediatric cancer population is cognitive behavioral therapy for insomnia, an effective method used in adult cancer survivors to improve sleep onset and decrease nighttime awakenings [135,149]. A similar, online version of cognitive behavioral therapy for insomnia is currently underway to determine its utility in pediatric survivors with insomnia [150]. 

Although the use of sleep medication in the non-cancer pediatric population is common, its use and safety in the pediatric cancer population is less studied [151]. Per the American Academy of Sleep Medicine, US Food and Drug Administration–approved medications that are known to be effective pharmacological treatments in adults with insomnia have not been studied in children [152,153,154]. For example, melatonin is an over-the-counter supplement that has shown some efficacy in treating sleep onset and circadian rhythm problems, including in obese survivors of childhood craniopharyngioma [69,115,155]. There is an obvious need to determine the efficacy and safety of sleep medications in cancer patients and survivors in the short- and long-term, including possible drug–chemotherapy interactions [156,157]. As Merz et al., detail, sleep medications in the pediatric cancer population may inadvertently perpetuate sleep disturbances by not treating the root cause, such as altered circadian rhythms, poor sleep hygiene, maladaptive sleep habits and behaviors, and a non-conducive sleep environment [106]. Moreover, the researchers explain that pediatric patients who habitually use sleep aids during treatment and survivorship may be at risk of suppressing their natural sleep cycle and falsely assume that medication is necessary to sleep. 

**Figure 2:**
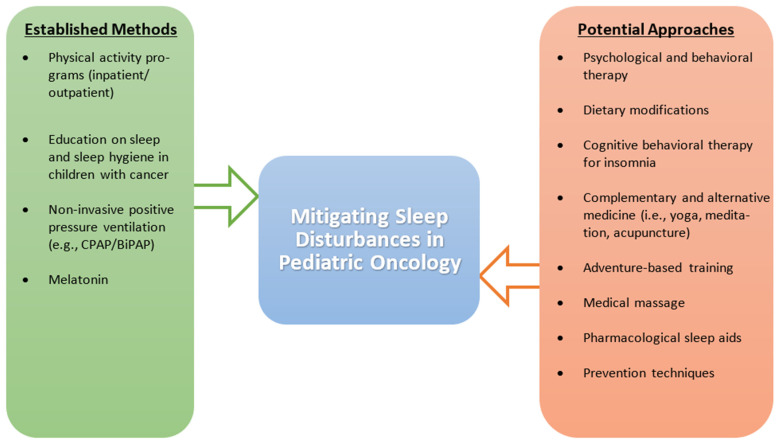
Established and potential management strategies to address sleep disturbances in pediatric cancer patients. The figure compares established methods that have been found to positively impact sleep to potential methods that require more investigation, specifically in the pediatric cancer population. Abbreviations: BiPAP, bi-level positive airway pressure; CPAP, continuous positive airway pressure.

Considering that EDS and SDB are significant contributors to sleep issues in the pediatric cancer population, there is a need to adopt treatment modalities for these sleep disturbances. Methods such as weight loss, positive pressure therapy, and adenotonsillectomy have been shown to provide relief from OSA, a form of SDB, in the general population [158,159,160,161]; because of their wide acceptance, these methods should be explored in the pediatric cancer population. Overall, there is an apparent need to focus on the safety and efficacy of pharmacological and non-pharmacological interventions in treating sleep disturbances in the pediatric cancer population [134]. Outside of physical activity, the current literature is inconclusive on medication and supportive care techniques for pediatric cancer patients, leaving a significant portion of this population vulnerable to the downstream effects of disturbed sleep. The various management strategies used to address sleep disturbances in pediatric cancer patients are described in Figure 2. 

## 8. Future Directions and Limitations

**Figure 3:**
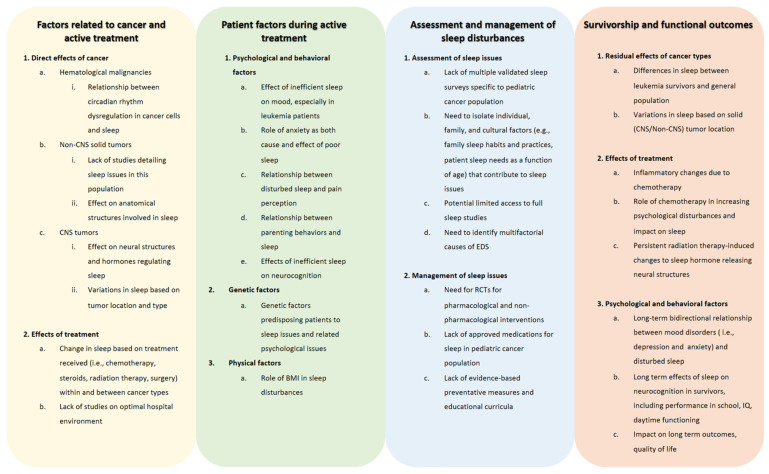
Gaps in knowledge on sleep disturbances in children with cancer. The figure describes areas where there is a need for further investigation in regard to sleep issues, their assessment and management, and their causes and sequalae during and after treatment, including many years into survivorship. Abbreviations: BMI, body mass index; CNS, central nervous system; EDS, excessive daytime sleepiness; IQ, intelligence quotient; RCT, randomized controlled trial.

Although nearly half of pediatric cancer patients and survivors experience disturbed sleep, there are significant gaps in our understanding of the role cancer plays in sleep issues and how to diagnose, prevent, and manage sleep problems. As described throughout this review, there are gaps in the literature in describing sleep issues due to the direct effects of cancers, such as the relationship between leukemia and circadian gene expression and the impact on sleep, as well as the direct role that solid tumors play in leading to disturbed sleep [45,46,49,52]. When describing a lack of the literature in patient characteristics that are affected by sleep issues during active treatment, there is a need to further describe the effect of inefficient sleep on mood and the bidirectional relationship of anxiety and sleep [63,106,107]. Moreover, while there are genetic factors that have been elucidated in terms of the genes that predispose a pediatric patient to further sleep issues and associated mood disorders, more work is needed to clarify genes already implicated and to discover other genetic variations as well [137]. When determining the best standard of practices to diagnose sleep disturbances, further studies are needed to guide clinicians in using validated instruments, such as the PROMIS surveys, and to standardize the use of modalities that assess sleep quality in pediatric cancer patients and survivors, including polysomnography, actigraphy, and various sleep questionnaires. These tools can then be utilized throughout treatment and survivorship to follow patients’ progression. In terms of treating sleep issues, data on both pharmacological and non-pharmacological modalities are severely lacking in pediatric cancer treatment and survivorship populations. Preliminary studies of sleep hygiene education targeted at children and parents, physical activity, and supportive care methods, such as massage, may serve as a springboard to broader and well-designed clinical trials to better understand the application of various interventions, given the multiple potential etiologies for sleep disturbances in this population [106,138,139,142]. Consequently, randomized controlled trials in the pediatric cancer population could help us determine the efficacy and safety of sleep medications, such as melatonin and sedating agents; the timing and intensity of sleep education; modalities of psychological interventions; and lifestyle interventions to ameliorate sleep difficulties. Sleep interventions that have been studied in the general pediatric population may serve as models for studying effective therapies to improve sleep in pediatric cancer patients and survivors. Further research is also needed to determine how to prevent maladaptive sleep changes. Determining how genetics, obesity, pain tolerance, treatment paradigms, cancer type, patient and family psychosocial characteristics, and sleep-related parenting behavior predispose patients to poor sleep may help us make progress toward that goal. A better understanding of etiological factors may lead to highly personalized intervention paradigms. When evaluating the gaps in the literature in regard to the long-term effects of the cancer diagnosis and its associated treatment on sleep, further research is needed to determine the longer-lasting effects of the malignancy and treatment on areas such as persistent inflammatory response and damage to structures of the brain. Moreover, similar to pediatric cancer survivors, larger prospective studies would assist in determining the psychological of a cancer diagnosis on sleep and the effects of sleep issues on the mood and cognitive outcomes of survivors. These gaps are further detailed in Figure 3. 

Considering that our literature review represented a scoping review, we identified limitations that place the results of our study in context. The discovery of the literature that we commented on was not based on a systemic methodology, and, thus, we did not evaluate the quality of the publications cited, despite ensuring that the papers included were published in peer-reviewed journals. Since the literature was researched manually through a database, e.g., PubMed, we may have missed certain studies on sleep disturbances in pediatric cancer patients, and our review is prone to selection bias. Moreover, due to the nature of the scoping review, the search for the literature required multiple different searches in the databases, thus increasing the variability of the literature cited. 

## 9. Conclusions

In pediatric cancer patients, sleep disturbances are common and multifactorial and have significant effects on the quality of life during treatment and survivorship. Regardless of cancer type, sleep changes, such as daytime sleepiness, insomnia, increased sleep onset time, and poor sleep quality, are common in pediatric cancer patients and survivors. The disease, treatment, home environment, and psychological predisposition of the patient play a role in the development and maintenance of sleep disturbances. While all children with cancer are at risk for sleep disturbances and the resulting deleterious effects on daily functioning, survivors of CNS tumors are affected the most significantly. 

Ultimately, improved sleep may be an essential component to enhancing short- and long-term functional, health, and quality-of-life outcomes of pediatric cancer patients and adult survivors. Improved sleep may be essential to reaching these goals. Increased research into sleep changes in pediatric cancer patients and survivors may help us develop diagnostic and treatment guidelines for general and oncology practitioners.

## Figures and Tables

**Table 1:** Summary of selected sleep-reporting tools and their utility in assessing sleep disturbances.

Sleep Reporting Tools	Form of Assessment	Population	Uses
Children Sleep Habits Questionnaire [122]	35-item, parent questionnaire, analyzes 8 different sleep domains	Parent-reported questionnaire: ages 4–10 years	Broadly assesses sleep disturbances with behavioral and medical causes. Evaluates bedtime resistance, sleep onset delay, sleep duration, sleep anxiety, nighttime awakening, parasomnia, SDB, and daytime sleepiness.
Sleep Disturbances Scale for Children [123]	26-item questionnaire that analyzes 5 different sleep domains	Parent- and self-reported: ages 3–16 years [125]	Categorizes the general type of sleep disturbance experienced. Assesses sleep initiation and maintenance disorders, arousal disorders, sleep–wake transition disorders, excessive somnolence, and sleep hyperhidrosis.
Epworth Sleepiness Scale for Children and Adolescents [126]	8-question survey	Modified scale for self-reporting: ages 12–18 years	Measures the effects of daytime sleepiness on adolescents’ physical and mental health, including effects on school performance.
Pediatric Daytime Sleepiness Scale [127]	8-question survey	Self-reported survey validated for children and adolescents in middle school: ages 11–15 years	Determines severity of daytime sleepiness and effects on outcomes in school performance. Correlates daytime sleepiness with changes in mood.
Patient-Reported Outcomes Measurement Information System Pediatric Sleep Disturbance and Sleep-Related Impairment item banks [124,128]	2 portions: 15-item questionnaire assessing sleep disturbances, 13-item questionnaire assessing sleep-related impairment	Self-reported version: ages 8–17 years; parent-reported version: ages 5–17 years	Assesses difficulties in falling and staying asleep and daytime sleepiness and their effects on daytime functioning.
Children’s Report of Sleep Patterns [129]	60-item questionnaire with 3 domains: Sleep Patterns, Sleep Hygiene Index and Sleep Disturbance scale	Self-reported questionnaire: ages 8–12 years	Use 3 domains, collectively or independently, to determine the source of sleep issues (sleep habits prior to bedtime, sleep patterns, and sleep disturbances).
Pittsburgh Sleep Quality Index [130]	19 sleep items analyzing sleep quality, assessed in healthy and non-healthy adults	Self-reported questionnaire initially designed for adults, with no pediatric-specific form Has been used in the pediatric cancer population [8,43]	Measures general sleep quality, taking into account sleep duration, sleep latency, daytime issues due to ineffective sleep, and use of sleep medication.
Sleep Diary [118]	Subjective tool to record nightly sleep information	Self- or parent-reported: all pediatric age groups	Follows a wide array of comprehensive data for each night’s sleep: duration of sleep, sleep onset latency, nighttime awakenings, and bedtime behavior. Preferred over questionnaires for further details on sleep–wake cycles. Most accurate when combined with actigraphy.
Actigraphy [131]	Wristwatch-like device used in the outpatient setting	All pediatric age groups	Assesses sleep–wake information, such as sleep onset, total sleep time, and nighttime awakening. Most accurate when combined with a sleep diary.
Polysomnography [132]	Diagnostic tool for the evaluation of sleep-disordered breathing, especially OSA	All pediatric patients; more data are needed for patients <6 months [133]	Gold standard for diagnosing sleep-disordered breathing and establishing non-invasive positive pressure ventilation. settings for therapy of OSA Can also be used to diagnose limb movement disorders.
